# Self-Management Support Program for Patients With Cardiovascular Diseases: User-Centered Development of the Tailored, Web-Based Program Vascular View

**DOI:** 10.2196/resprot.6352

**Published:** 2017-02-08

**Authors:** Saskia Puijk-Hekman, Betsie GI van Gaal, Sebastian JH Bredie, Maria WG Nijhuis-van der Sanden, Sandra van Dulmen

**Affiliations:** ^1^ Radboud university medical center Radboud Institute for Health Sciences IQ healthcare Nijmegen Netherlands; ^2^ Institute of Nursing HAN University of Applied Sciences Nijmegen Netherlands; ^3^ Radboud university medical center Department of General Internal Medicine Nijmegen Netherlands; ^4^ Radboud university medical center Radboud Institute for Health Sciences Department of Primary and Community Care Nijmegen Netherlands; ^5^ NIVEL (Netherlands institute for health services research) Utrecht Netherlands; ^6^ Faculty of Health Sciences University College of Southeast Norway Drammen Norway

**Keywords:** intervention mapping, eHealth, self-management, cardiovascular diseases, tailoring, nursing care, chronic secondary care, outpatients, early RCT

## Abstract

**Background:**

In addition to medical intervention and counseling, patients with cardiovascular disease (CVD) need to manage their disease and its consequences by themselves in daily life.

**Objective:**

The aim of this paper is to describe the development of “Vascular View,” a comprehensive, multi-component, tailored, Web-based, self-management support program for patients with CVD, and how this program will be tested in an early randomized controlled trial (RCT).

**Methods:**

The Vascular View program was systematically developed in collaboration with an expert group of 6 patients, and separately with a group of 6 health professionals (medical, nursing, and allied health care professionals), according to the following steps of the intervention mapping (IM) framework: (1) conducting a needs assessment; (2) creating matrices of change objectives; (3) selecting theory-based intervention methods and practical applications; (4) organizing methods and applications into an intervention program; (5) planning the adaption, implementation, and sustainability of the program, and (6) generating an evaluation plan.

**Results:**

The needs assessment (Step 1) identified 9 general health problems and 8 determinants (knowledge, awareness, attitude, self-efficacy, subjective norm, intention, risk perception, and habits) of self-managing CVD. By defining performance and change objectives (Step 2), 6 topics were distinguished and incorporated into the courses included in Vascular View (Steps 3 and 4): (1) Coping With CVD and its Consequences; (2) Setting Boundaries in Daily Life; (3) Lifestyle (general and tobacco and harmful alcohol use); (4) Healthy Nutrition; (5) Being Physically Active in a Healthy Way; and (6) Interaction With Health Professionals. These courses were based on behavioral change techniques (BCTs) (eg, self-monitoring of behavior, modeling, re-evaluation of outcomes), which were incorporated in the courses through general written information: quotes from and videos of patients with CVD as role models and personalized feedback, diaries, and exercises. The adoption and implementation plan (Step 5) was set up in collaboration with the members of the two expert groups and consisted of a written and digital instruction manual, a flyer, bimonthly newsletters, and reminders by email and telephone to (re-)visit the program. The potential effectiveness of Vascular View will be evaluated (Step 6) in an early RCT to gain insight into relevant outcome variables and related effect sizes, and a process evaluation to identify intervention fidelity, potential working mechanisms, user statistics, and/or satisfaction.

**Conclusion:**

A comprehensive, multi-component, tailored, Web-based, self-management support program and an early RCT were developed in order to empower patients to self-manage their CVD.

**Trial Registration:**

Nederlands Trial Register NTR5412; http://www.trialregister.nl/trialreg/admin/rctview.asp?TC=5412 (Archived by WebCite at http://www.webcitation.org/6jeUFVj40)

## Introduction

Cardio vascular disease (CVD) is the leading cause of death worldwide [1]. In 2012, 17.5 million people died of CVD, within which 7.4 million deaths were due to heart attacks and 6.7 million deaths resulted from strokes [1]. The incidence of CVD, and its consequences for chronic secondary care, continue to rise and place high demands on scarce health care resources [1].

CVD is predominantly caused by genetic and environmental factors, among which unhealthy lifestyle habits are the most important. Risk factors of CVD caused by atherosclerosis are divided into behavioral risk factors (physical inactivity, an unhealthy diet rich in salt, fat, and calories, tobacco use, and the harmful use of alcohol), metabolic risk factors (hypertension, diabetes, raised blood lipids, and overweight/obesity), and other risk factors (eg, advancing age, gender, stress, and depression). Since all risk factors interact with each other and play a key role in decreasing the process of atherosclerosis, they must be considered in the treatment of CVD and secondary prevention [1].

Health and social care services support patients with chronic diseases by providing specialized staff, medicines, and equipment to control symptoms. In addition to medical treatment and counseling, patients need to manage CVD and its consequences in daily life by themselves [2]. Self-management assumes that patients feel confident dealing with the symptoms, treatment, lifestyle changes, and physical and psychological consequences related to illness [3,4]; however, many patients do not feel confident enough to manage their CVD [5-7]. Self-management programs that encourage patients to identify problems, barriers and support, generate solutions, and develop and monitor long- and short-term goals may therefore help patients in actively controlling and improving their own condition [3,8].

Self-management programs have the potential to decrease the load on health and social services, to lower the high costs of chronic care, and to improve patient quality of life. Self-management in chronic care appears to be effective, especially when focusing on behavioral change, supporting self-efficacy, and implemented in wider initiatives (eg, information provision, online peer support, monitoring symptoms with technology, and psychological and behavior change interventions) [2]. Although the effectiveness of traditionally delivered, face-to-face, self-management programs is proven [9-11], the major part of their care depends on a patient’s self-management skills, for which Web-based programs are expected to be helpful [12]. However, Web-based programs demonstrate varied and inconsistent effectiveness due to design limitations and lack of power [12]. Only a few studies suggest that these programs for CVD are effective in risk factor reduction, secondary prevention, clinical outcomes, reductions in hospital admissions, and mortality [12-15].

To our knowledge, there are no comprehensive, multi-component, Web-based, self-management support programs that focus on behavior change and support self-efficacy in secondary care patients with CVD. Within such programs, the tailoring of the content to a patient’s profile is important in order to increase their level of understanding, information recall, and adherence to lifestyle interventions [16]. Therefore, we systematically developed a tailored, Web-based, self-management program that aims to change behavioral and metabolic risk factors and increase self-management behavior, based on the perceived problems and (support) needs of patients in managing secondary prevention after CVD.

The aim of this paper is to describe (1) the development of the “Vascular View” (“Vaat in Zicht” in Dutch) program for patients with CVD, according to six steps of the intervention mapping (IM) framework [17]; and (2) the plans we have made to systematically evaluate Vascular View in an early randomized controlled trial (RCT). The early RCT will test the viability of a larger clinical trial, including the ability to recruit a relatively small number of patients and to explore the potential efficacy and effectiveness of the intervention under study [18].

## Methods

IM is an iterative, 6-step process for developing theoretically-based behavior change interventions [17]. The steps are (1) conducting a needs assessment among end-users according to the Predisposing, Reinforcing, and Enabling Constructs in Educational/Environmental Diagnosis and Evaluation (PRECEDE) model; (2) creating matrices of change objectives; (3) selecting theory-based intervention methods and practical applications; (4) organizing methods and applications into an intervention program; (5) planning the adaption, implementation, and sustainability of the program; and (6) generating an evaluation plan.

Throughout the entire development process for the Vascular View program, two expert groups (one of patients and one of professionals) explored and discussed, at each IM step, the specific issues (disease and treatment) that may influence self-management. The groups met up to three times to ensure that the program would be tailored to the perceived problems and needs that are important to patients with CVD and their health professionals. The patient expert group consisted of 1 female and 5 males with CVD (stroke and/or cardiac events and/or peripheral artery disease). The professional expert group included a medical specialist in general and vascular medicine, a neurology nurse, a cardiology nurse, a vascular surgery nurse, a psychologist, a dietician, and a physical therapist. Two researchers (SPH and BvG) participated in both expert groups and SPH chaired each meeting. Before the start of each expert group meeting, the members were asked to prepare by reading information and finishing assignments on the themes of the meeting. The meetings were supported by PowerPoint presentations and the researchers emphasized the importance of the opinions and contributions of each member. This resulted in valuable discussions and agreements at the end of each meeting, which were audio recorded. After the meetings, the recordings were transcribed verbatim by a student. Analysis of all meetings took place by thematically verifying researcher’s (SPH) notes with the typed results of the audio recordings, in summary.

### Step 1: Conducting a Needs Assessment

The needs assessment focused on identifying perceived problems and (support) needs in the self-management of CVD using the Predisposing, Reinforcing, and Enabling Constructs in Educational/Environmental Diagnosis and Evaluation (PRECEDE) model [19]. First, we sampled the literature for perceived problems related to the self-management of CVD [5-7,20-23]. Second, the problems identified were extracted by the researchers and discussed with and prioritized by the members of both expert groups during one meeting to generate a complete list. The researchers then clustered the prioritized perceived problems into general health problems. In addition, they selected the underlying behaviors and environmental conditions of the health problems and the corresponding determinants (factors which were linked to the underlying behaviors and environmental conditions) from the literature. Finally, in two meetings, the members of the two expert groups discussed the selection and determined the most important and modifiable underlying behaviors and determinants required to self-manage CVD.

### Step 2: Creating Matrices of Change Objectives

The aim of creating matrices of performance and change objectives was to provide a translation from needs to content in Vascular View. Behavioral and environmental conditions selected were related to self-management of the problems identified in Step 1. These were subdivided into performance objectives and combined with the important and modifiable determinants of the behavioral and environmental conditions based on the Integrated Change Model 2.0 (I-Change model 2.0) [24], and described in matrices of change objectives. For example, the performance objective “CVD patients understand their disease and the accompanying symptoms and consequences” was combined with the determinant “knowledge,” which resulted in the change objective “CVD patients know their disease and accompanying symptoms and consequences.” In one meeting with both expert groups, the identified performance and change objectives were then pointed out and validated by the members of the two expert groups to ensure the designed matrices did indeed address the problems of patients with CVD. Finally, in the same meeting, the two expert groups were asked to prioritize the topics that should be incorporated in the Vascular View program.

### Step 3: Selecting Theory-Based Intervention Methods and Practical Applications

The purpose of Step 3 was to select theory-based intervention methods and practical applications based on the former steps. The Coding Manual for Behavior Change Techniques from de Bruin and colleagues [25] was used to select the methods used to change the sub-behaviors of self-management and the corresponding determinants from Step 2. This manual provides behavior change techniques (BCTs) in general (eg, tailoring) and on determinant level. The IM framework identifies the parameters under which a given technique is most likely to be effective [17,25]. Two researchers (SPH and BVG) determined relevant BCTs based on the selected determinants and translated these techniques into practical applications, such as text, personal stories, exercises, diaries, and videos. In one meeting, the selected BCTs and practical applications were then discussed with the members of the two expert groups to decide whether the techniques fit the target group.

### Step 4: Organizing Methods and Applications Into an Intervention Program

The aim of Step 4 was to develop and pretest the program components and materials of Vascular View. The program outcomes, the selected BCTs, and the practical applications from Steps 2 and 3 were starting points for the development and pretest of the program. All members of the two expert groups were asked what the ideal Vascular View program should look like, in line with the message (eg, tailoring, text, video, and exercise), the channel (Internet), and the information sources (eg, websites, patient forum). All patients from the expert group were also asked for the most valuable perceived advices that they received during their treatment and rehabilitation, how these advices looked like, and from whom these messages came. All health professionals were asked how they adapted their advice given to a patient and how they validated this adaptation. The research group prepared blueprints for the production of the different program materials and discussed this within the two expert groups in one meeting. Based on the outcomes of this meeting, the final content (ie, text, personal stories, diaries, and videos) was determined and developed by the research group and incorporated into the Vascular View program by Mind District Development BV (The Netherlands), the information and communication technology (ICT) partner. Finally, the program was pretested by the members of both expert groups individually and their feedback was gathered by open-ended questionnaires in which the members could describe their comments on each page. All patients and health professionals were asked to use the program for one week and give feedback about the importance and comprehensibility of the information, quotes, pictures, videos, diaries, and exercises, and their views of the layout of Vascular View and whether it was user-friendly. All of the feedback was collected, structured per training session and specific page, and incorporated into the final program. See also the results of Step 4.

### Step 5: Planning for Program Adoption, Implementation, and Sustainability

The focus of Step 5 was to make an implementation plan for Vascular View during the planned evaluation study. The adoption and implementation of Vascular View started in the development process with the involvement of patients and professionals by taking their expertise into account. In addition to their role in the development of the program, the two expert groups played an important role in the determination of facilitators for, and barriers to, the adoption and implementation of this intervention. For example, all members were asked, via questionnaires, what facilitates dissemination and exposure (eg, logging in for the first time and subsequent logging in), in order to complete the different courses of Vascular View. The researchers also determined performance objectives for a patient’s first visit and re-visits to Vascular View and combined these with the relevant determinants into change objectives, and discussed these results in one meeting with the members of the two expert groups. The findings of dissemination and exposure were incorporated in the final Vascular View program. The performance and change objectives, and the corresponding determinants, were included in the adoption and implementation plan for Vascular View.

### Step 6: Generating an Evaluation Plan

The objective of the final step of IM was to design an evaluation study for Vascular View by conducting an early RCT to gain insight into relevant outcome variables and related effect sizes, and to perform a process evaluation to identify intervention fidelity, potential working mechanisms, and user satisfaction [26,27].

In this phase, we selected relevant measurable outcome variables related to the objectives of the Vascular View program, the defined performance outcomes, and whether the specific tailoring to changes in self-management behavior conformed to the applied I-Change Model 2.0. The process evaluation also includes a check of intervention fidelity (adherence to the program as proposed), incorporating outcomes compliant with the performance objectives and determinants as expressed in the matrix of change objectives in IM Step 2, as well as interviews with participants (program users and non-users) of the study, audio recordings of nursing visits with participating patients, and focus group interview(s) with health professionals.

## Results

The involvement of the members of both expert groups for each IM step during the development of Vascular View is described in Table 1.

**Table 1:** Involvement of stakeholders in the development of Vascular View.

Step	Intervention mapping		Meetings, n	Patients, n (gender)	Health professionals, n
1	Needs assessment		3	6 (1 female, 5 males)	7^a^
2	Objectives		1	5 (males)	3^b^
3	Theory		1	6 (1 female, 5 males)	6^c^
4	Intervention program		1	4 (males)	5^d^
		Pretest Vascular View	N/A	6 (1 female, 5 males)	7^a^
5	Implementation		1	4 (males)	5^d^
6	Evaluation		1	4 (1 female, 3 males)	4^e^

^a^Neurology nurse, cardiology nurse, vascular surgery nurse, psychologist, dietician, physical therapist, and medical specialist in general and vascular medicine

^b^Neurology nurse, vascular surgery nurse, and medical specialist in general and vascular medicine.

^c^Neurology nurse, cardiology nurse, vascular surgery nurse, psychologist, dietician, medical specialist in general and vascular medicine.

^d^Neurology nurse, cardiology nurse, vascular surgery nurse, dietician, medical specialist in general and vascular medicine.

^e^Neurology nurse, cardiology nurse, vascular surgery nurse, medical specialist in general and vascular medicine.

### Step 1: Needs Assessment

**Figure 1:**
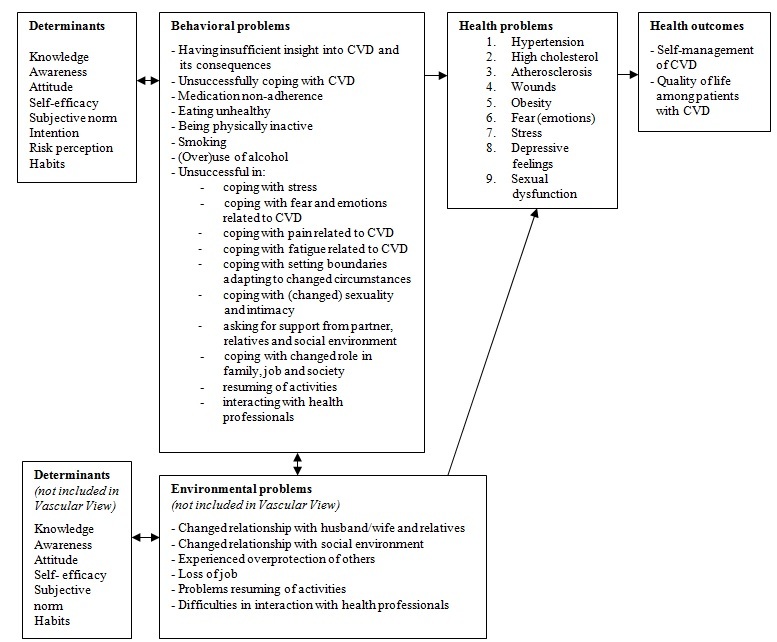
Contextualized Predisposing, Reinforcing, and Enabling Constructs in Educational/Environmental Diagnosis and Evaluation (PRECEDE) model as a logic model for the needs assessment of self-managing cardiovascular disease.

A literature search and three meetings with the two expert groups (Table 1) elicited perceived and experienced problems related to self-managing CVD [5-7,20-23]. The comprehensive results of the first meeting were clustered into 9 general health problems and the accompanying important symptoms (fatigue and pain), which were applied specifically to self-management in CVD (Figure 1). 

The second and third meetings provided the selected underlying behaviors and environmental conditions of these health problems and symptoms, which varied from having insufficient insight into CVD to having an unhealthy lifestyle, and being unsuccessful in setting boundaries, or inadequate interaction with health professionals (Figure 1). The following corresponding determinants of these underlying behaviors and environmental conditions were extracted from the literature: (1) knowledge, (2) awareness, (3) attitude, (4) self-efficacy, (5) subjective norm, (6) intention, (7) risk perception, and (8) habits. Because the Vascular View program will focus on patients’ behavior, we did not explicitly involve environmental factors and corresponding determinants. For example, the health problem “obesity” is related to the behavior “eating unhealthy” with the corresponding determinants knowledge (patients know what healthy nutrition is) and self-efficacy (patients believe they are able to eat healthy).

### Step 2: Matrices of Change Objectives

**Figure 2:**
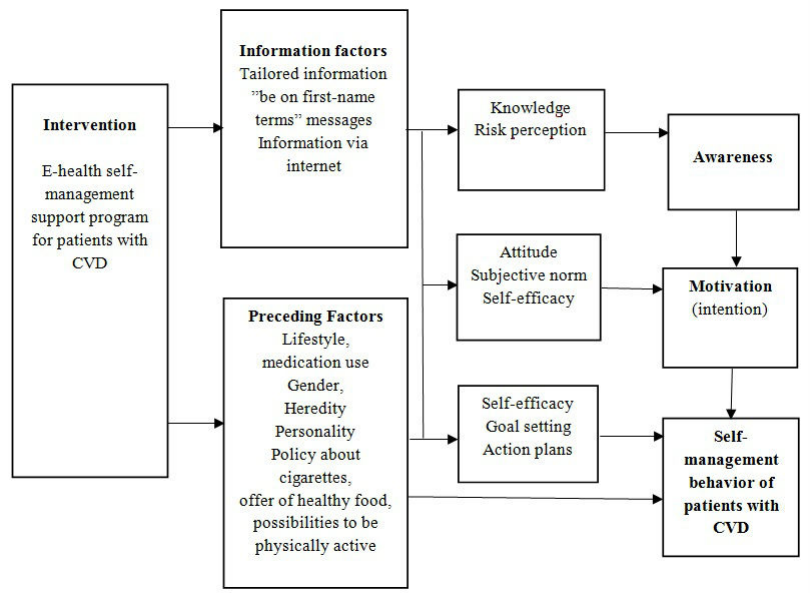
Applied Integrated Change Model (I-Change) 2.0 for increasing the self-management behavior for cardiovascular disease.

We selected the three most important and modifiable determinants of self-managing CVD from the I-Change Model 2.0 [24]: awareness, motivation, and action. A patient should have accurate knowledge and risk perception to be aware that self-managing CVD is a possibility and a choice. A patient’s motivation (intention) to increase self-management behavior depends on the patient’s attitude (perceived pros and cons), beliefs about subjective norms (reactions and expectations of important others), and self-efficacy expectations (perceived ability to perform self-management behavior). Action is needed to increase self-management behavior, which is determined by self-efficacy, goal setting, action-planning, and the development of the skills needed for CVD self-management. Finally, we defined predisposing factors, such as lifestyle, gender, heredity, personality, policy about cigarettes, supply of healthy food, and potential to be physically active. The applied I-Change Model 2.0 for increasing self-management behavior in CVD patients is shown in Figure 2.

In one meeting, the members of the two expert groups (Table 1) validated the designed matrices of performance and change objectives and prioritized the following topics of the performance and change objectives: (1) lifestyle (nutrition, physical activity, smoking and use of alcohol); (2) setting boundaries; (3) medication adherence; (4) emotions (fear); and (5) interaction with the health professional.

Multimedia Appendices 1 and 2 show matrices in which the selected most important and modifiable determinants were added to the performance objectives of each topic according to the I-Change Model [24], and combined with change objectives.

### Step 3: Theory-Based Intervention Methods and Practical Applications

For each selected determinant of self-management behavior (eg, knowledge, risk perception, awareness, attitude, self-efficacy, subjective norm, intention, and habits), the BCTs were selected [25] and discussed with both expert groups (Table 1) during one meeting. According to the Coding Manual for Behavior Change Techniques [25], a patient’s knowledge will be influenced by providing general information and increasing the memory and/or understanding of transferred information. Awareness will be influenced by self-monitoring and delayed feedback of behavior (eg, by providing an overview of recorded, self-reported, behavior) (see Multimedia Appendix 3 for a screenshot of a diary of Vascular View) whereas risk communication will change a patient’s risk perception. Intention will be modified by specific goal setting and review of general and/or specific goals (see Multimedia Appendix 4 for a screenshot of a step-by-step plan of Vascular View), and modeling, setting graded tasks, and goal setting will influence self-efficacy (see Multimedia Appendix 5 for a screenshot of role models in Vascular View). Attitude will be modified by a reevaluation of outcomes, self-evaluation, persuasive communication, and belief selection and providing information about peer behavior and mobilizing social norm will change subjective norm. The maintenance of a patient’s behavior change will be influenced by formulating goals for maintenance of behavior and relapse prevention. Finally, it is suggested that a behavior change program in which the content and exercises can be adapted to a patient’s profile is most likely to be effective [11,12]. Therefore, personalized tailoring is integrated in Vascular View in a number of ways (described below).

In translated these BCTs to practical applications, the parameters under which a given technique is most likely to be effective were taken into account. For example, self-reevaluation can use feedback and confrontation; however, raising awareness must be quickly followed by increase in problem-solving ability and self-efficacy. Multimedia Appendix 6 shows how we developed the practical applications from determinants (awareness, intention, and self-efficacy), BCTs, and parameters [17,24].

### Step 4: Intervention Program

In one meeting, the two expert groups (Table 1) emphasized the use of brief sentences, dynamic content (different videos, quotes, exercises, diaries, pictures/cartoons), and to write on first-name terms in the Web-based program. It was also found that the program should enable patients to write down their questions (question prompt sheet), which can be taken to a consultation with their health professional. The program should enable a patient to make a printable, personal plan for improvement. The meetings with the two expert groups and the results of IM Steps 1 to 3 resulted in the current Vascular View program. The program starts with a welcome for every patient, in which the courses (Coping with CVD, Setting Boundaries in Daily Life, Lifestyle, Healthy Nutrition, Being Physically Active in a Healthy Way, and Interaction with Health Professionals) are explained (Multimedia Appendix 7). A patient can randomly visit and complete every individual course. In the welcome section, patients fill in a short questionnaire, which guides their choice of preferred courses.

The course “Coping with CVD” addresses the determinants knowledge, awareness, risk perception, attitude, self-efficacy, subjective norm, intention, and action plans. The patient learns about dealing with CVD and its impact on daily life (eg, medication adherence, sexuality, emotions, fatigue, and pain), relatives, social environment, and resuming activities.

In the course “Setting Boundaries,” patients learn to clearly communicate with their social environment (eg, relatives, colleagues) about perceived boundaries in daily life and in resuming activities with respect to their current ability. This course addresses the determinants knowledge, awareness, attitude, self-efficacy, and subjective norm.

The course “Lifestyle” addresses the determinants knowledge, awareness, attitude, self-efficacy, subjective norm, intention, habits, and skills, and consists of information about a healthy lifestyle (eg, risk information about tobacco and alcohol use in CVD, and their health), and information to support abstention from tobacco and alcohol use.

In the courses “Healthy Nutrition” and “Being Physically Active in a Healthy Way,” patients gain insight into what they eat and drink, how physically active they are, and how to change their habits to healthier ones, step by step. These courses address the determinants knowledge, awareness, attitude, self-efficacy, subjective norm, intention, habits, and skills.

The course “Interaction With Your Health Professional” teaches patients to effectively communicate with the health professional (eg, preparing a consultation, asking questions, sharing worries). This course addresses the determinants knowledge, awareness, attitude, self-efficacy, and subjective norm.

The sessions in the courses are personalized and are supported by written information, quotes, pictures, videos, diaries, and exercises. In the courses “Healthy Nutrition” and “Being Physically Active in a Healthy Way,” two case studies involving two imaginary patients (a man and a woman) support the information and exercises offered through the sessions and courses (Multimedia Appendix 7).

In all courses of Vascular View, tailoring was done using variables or factors related to behavior change (such as stage of change), or to relevance (such as culture or socioeconomic status) [17,25]. A patient is able to choose preferred courses in the welcome section. The “Lifestyle” course also contains a short questionnaire about nutrition, weight, height, physical activity, and tobacco and alcohol use, which is transformed into tailored, written feedback including red (unhealthy behavior) and green (healthy behavior) emoticons, followed by tailored guidance to undertake different courses of Vascular View (Setting Boundaries, Healthy Nutrition, and Being Physically Active in a Healthy Way). The healthy nutrition advice is also tailored to age using an algorithm (ages 19 to 50 years, 51 to 70 years, and greater than 70 years), to fit the age-based Dutch guidelines for a healthy diet [28]. Tailoring takes place according to the detail of the information (“read more” options in all of the courses). To support the change processes in patients, we used the short form version of the Scale for Interpersonal Behavior (s-SIB) [29] and motivational interviews [30].

The feedback of the pretest by all patients and health professionals of both expert groups (Table 1) consisted of usability issues (eg, problems with logging in) and feedback on text issues (patronized texts, typological and layout errors, the amount of “passive” texts). This resulted in the adaption of Vascular View (eg, text of all courses were shortened, medical information was rewritten, the Healthy Nutrition and Being Physically Active courses were rewritten in a more interactive way, and typological and layout errors were removed) and different implementation strategies (see also Step 5). For example, we simplified the user manual by reducing text and including screenshots of Vascular View to guide the use of the program.

### Step 5: Program Adoption, Implementation, and Sustainability

As a result of the first 4 Steps and one meeting with both expert groups (Table 1), we developed different implementation strategies for patients as well as health professionals. For patients, we determined that motivation and perceived personal relevance on the first visit were the most important factors in the dissemination of, and exposure to, Vascular View. Important facilitators of re-visiting Vascular View were tailored feedback, new, relevant, and reliable information, a user-friendly program, reminders to re-visit the program and repeating personal points of improvement, and the possibility of email contact [31,32]. Therefore, the following implementation strategies were developed for patients: patients receive a written instruction manual for Vascular View and the diaries (nutrition and physical activity) are made available before receiving the codes to log in. Upon the start of the program, a digital flyer is sent to patients, which promotes the benefits of using the program and contains information about contact persons. Patients receive a code to log in when they receive the flyer. The patients who do not visit the program within one month receive an email reminder. If patients do not log in after another week, they receive a phone call from a researcher. Patients also receive an email when they visit the program but do not finish a task or a course. Finally, every 2 months a newsletter is sent to all participants to informally remind them of Vascular View with news about the program, the ongoing research project, and contact details in case of questions or problems with logging in.

The members of the two expert groups also emphasized the importance of being motivated to use Vascular View by their health professionals, whether or not this was supported by a brochure. They added that health professionals should also be interested in the progress of a patient’s self-management behavior, and be able to answer questions during the study in order to show their involvement.

The key persons in adoption and implementation are the medical specialists in Internal and Vascular Medicine, and the nurse specialists in Neurology, Cardiology, and Vascular Surgery, because of their crucial roles in the recruitment of patients. They also brought Vascular View to the attention of patients during regular consultations across the study period by asking about the program. Before the program started, the nurse specialists were instructed in how to support a patient’s use of Vascular View.

### Step 6: Evaluation Plan

#### Design

A mixed-methods study design will be conducted with an early RCT and a process evaluation to (1) evaluate the potential effectiveness and effect size of Vascular View; (2) identify the outcome measures most likely to capture potential patient benefit; and (3) evaluate continued participation or withdrawal from Vascular View. The early RCT and the process evaluation will be performed at four outpatient clinics in one university hospital in the Netherlands (Internal Medicine, Cardiology, Neurology, and Vascular Surgery).

#### Participants

We will recruit 400 to 600 potential participants diagnosed with CVD, to allow the participation of 200 patients. Inclusion criteria are that patients (1) manifest atherosclerotic vascular disease, including ischemic heart disease, cerebrovascular disease (eg, stroke or transient ischemic attack), and peripheral artery disease (eg, claudication intermittens), or a combination; (2) are aged 18 years or older; (3) can speak, read, and understand the Dutch language; and (4) have access to a computer, the Internet, and an email account. Exclusion criteria are patients with (1) comorbidities, which may hinder the use of Vascular View, as defined by the medical specialist; and (2) psychiatric disorders.

#### Procedure

The medical specialists will inform patients about the content and aim of the study via an information letter. After signing the informed consent and completing the baseline questionnaire (T0), patients will be included and randomized to an intervention group (access to Vascular View for 1 year on top of usual care) and a control group (usual care). Randomization will take place at the individual level, will be stratified for diagnoses, and will be executed by a statistician who will use a computer program. Patient characteristics will be assessed at baseline via an online questionnaire (T0) and medical file research. Repeated measures will be conducted 6 months (T1) and 12 months (T2) after baseline. Semi-structured interviews will be performed with patients and focus group interview(s) will be conducted with health professionals at 12 months.

#### Ethical Approval

This study has been improved by the Medical Ethical Research Committee of Arnhem - Nijmegen, Nijmegen, the Netherlands (registration number: 2015/1908), and is registered in the Netherlands Trial Registry (registration number: NTR5412).

#### Measurements

The quantitative data will be collected via an online questionnaire and medical file research. The online questionnaires consist of patient demographics and the following, described 11 measurements. Illness attribution will be assessed by one question in the Illness Perception Questionnaire (IPQ) [33]. The health-related quality of life survey (RAND-36) measures a patient’s general health status in 8 dimensions including physical and social functioning, role limitations (physical and emotional problems), mental health, vitality, and pain [34]. Self-management will be evaluated using Patient Activation Measurement (PAM-13) [35]. Self-efficacy will be assessed through self developed questions in which patients will be asked how confident they feel about self-managing CVD based on the program outcomes and corresponding determinants. Patient trust in their communication skills will be measured by the Perceived Efficacy in Patient-Physician Interactions (PEPPI-5) [36]. Patient attitudes to prescription medicine will be evaluated using the Beliefs about Medicines Questionnaire (BMQ) [37]. The Morisky Medication Adherence Scale (MMAS-8) will assess medication adherence [38]. Finally, lifestyle will be evaluated using the Fägerstrom Test for Nicotine Dependence [39] for tobacco use, the Alcohol Use Disorders Identification Test (AUDIT) [40] for testing harmful alcohol use, the International Physical Activity Questionnaire (IPAQ) [41] to identify a patient’s physical activity, and eating habits will be evaluated by the Dutch Healthy Diet Index [42]. We will sample biometrics from patient medical files, including blood pressure, BMI, etc.

#### Process Evaluation

To investigate the factors that influence user statistics, intervention fidelity, and user satisfaction in the Vascular View program, a process evaluation will be performed based on the framework of Saunders et al [27]. This is a comprehensive and systematic framework for developing a process-evaluation plan to evaluate the implementation of a targeted health promotion intervention, which comprises components including fidelity, dose (delivered and received), reach, recruitment, and context [27]. Data for the process evaluation builds on different sources. An extra questionnaire will be completed at T1 and T2, in which experiences and opinions of the written manual, use and non-use of the program, layout, content, exercises, and diaries of the program will be asked of patients in the intervention group. Data obtained from the Web-based program will be used, including login data (exposure and continued participation or dropping out), data about using different courses, sessions, and the use of the diaries. Qualitative interviews with users and non-users will be conducted to explore their experiences and satisfaction with Vascular View. Patients will be asked how Vascular View supported the self-management of their CVD, including coping with CVD and its consequences, setting boundaries, changing lifestyles (being physical active, eating healthily, cessation of smoking, and the harmful use of alcohol), and communicating with health professionals. Patients who do not log in, or log in only once, will be asked about their reasons for not visiting the Vascular View program and the barriers they perceived. Nursing visits with participating patients will be audio recorded and analyzed to explore the role of nurses and activities related to the introduction of Vascular View. Finally, focus group interview(s) with health professionals in chronic diseases will be conducted to determine their opinions of the adaptation and implementation of a Web-based program and their own role in this process and in patient treatment.

#### Analyses

Quantitative and qualitative analyses will be performed. Patient outcome measures will be treated as dependent and continuous variables and will be analyzed on the basis of intention-to-treat. All variables will be presented in percentages or in means and standard deviations of the sum-scores. Repeated measures analyses will be performed to explore effect sizes and the responsiveness of outcome measures. Multilevel analyses will be applied to compensate for the clustered nature of the data using mixed linear modeling techniques. The qualitative data gathered in semi-structured interviews and the focus group interviews will be analyzed using content analyses.

## Discussion

### Principal Findings

The aims of this paper are (1) to describe the development of the tailored, Web-based, self-management support program Vascular View for patients with CVD according to the 6 steps of the IM framework; and (2) to describe how the program will be evaluated. The 6 steps of IM provide a well-balanced processual guide for tailoring to the preferences and support needs of patients with CVD, by combining evidence and the perceived experiences of patients and their health professionals. A unique feature of the program is that it is based on the perceived problems of patients with CVD and that the corresponding determinants were combined with theory-based intervention methods into practical applications. This resulted in a Web-based program which may influence the relevant determinants—knowledge, awareness, attitude, self-efficacy, subjective norm, risk perception motivation (intention), habits, and maintenance—of self-management in patients with CVD by applying different methods through the courses. The Vascular View program was tailored to patient preferences, the level of elaborateness of the information, the content, factors related to behavior change, and relevance. To our knowledge, such a comprehensive, tailored, Web-based, self-management program for secondary care patients with CVD has not been previously developed; other programs have mainly focused on one or two risk factors or risk factor determinants only [12-15].

### Strengths and Limitations

A strength of the Vascular View program is the intensive cooperation of the two expert groups in every step of IM. The two expert groups ensured that the general information from the literature fitted the target group. Their knowledge, experiences, and visions led to the comprehensive, Web-based program developed.

Vascular View was developed for a heterogeneous population of patients with manifest CVD, including, among others, ischemic heart disease, cerebrovascular disease, and peripheral artery disease. This diversity in patients and diagnoses required a well-balanced approach from the Web-based program. To overcome this possible limitation, we tailored the content of the courses to patient diagnoses and interests by using short questionnaires before or during a course and “read more” options within the courses.

Despite the comprehensiveness of this Web-based program, Vascular View is limited in its influence over the different environmental factors of patients with CVDs. Different interpersonal (eg, partner, relatives, colleagues), organizational (eg, unavailability of accompanied exercise facilities by a health professional), community (eg, availability of work and income), and societal (eg, no smoking allowed in public buildings) factors were incorporated in Vascular View because the program aimed to increase self-management behavior and the way patients living in this (interpersonal, organization, community, and society) environment are able to cope, via the patients themselves. However, we do not know how these different environmental factors interact in order to change behavior [17]. In designing Vascular View, we made use of two highly relevant theoretical models [17,25]. For future studies, to prevent confusion, attention should be paid to the similarities and dissimilarities between the terminology and terms used in these different models. The composition of the patient expert group might be considered a limitation, because only 1 female patient as opposed to 5 males participated, which is not a representative reflection of the population. It might be considered as a limitation that we left out interventions on smoking and drinking behavior. Because of their availability, we give general information and provide links to already existing, highly effective programs. Finally, the pilot testing of Vascular View will take place in one university hospital in the Netherlands only. For an early RCT that aims to obtain insight into the relevant outcome variables and related effect sizes, such a mono-centered study is considered to be the most convenient [18,26].

### Conclusions

This paper describes the systematic development of the comprehensive, tailored, Web-based self-management support program Vascular View for patients with CVD, according to the IM framework. Vascular View aims to increase self-management behavior in patients with CVD by influencing the risk factors of CVD and through an improvement in quality of life, via a unique combination of tailoring, personalizing, and behavior change, by influencing determinants (knowledge, awareness, attitude, self-efficacy, subjective norm, risk perception, intention, and habits) of self-management behavior. The proposed early RCT will indicate whether these aims can be achieved. Furthermore, this feasibility study will give insight into relevant outcome variables and related effect sizes and allow us to identify any flaws or technical difficulties. This information will be used to set up a larger RCT. A process evaluation will identify, amongst other things, patient experiences in using the program and at which level the Vascular View program is helpful in supporting self-managed CVD. This information will be used in the future development of the Vascular View program.

## Appendix

Multimedia Appendix 1 Matrix of performance and change objectives for patients with cardiovascular disease (according to the boxes in the applied Integrated Change Model 2.0).

Multimedia Appendix 2 Matrix of performance and change objectives for patients with cardiovascular disease (according to the boxes in the applied Integrated Change model 2.0).

Multimedia Appendix 3 Screenshot of Vascular View ("Awareness").

Multimedia Appendix 4 Screenshot of Vascular View ("Intention").

Multimedia Appendix 5 Screenshot of Vascular View ("Self-efficacy").

Multimedia Appendix 6 Development of Vascular View from determinants to practical applications.

Multimedia Appendix 7 An overview of the 6 courses of Vascular View.

## Abbreviations

BCTs: behavior change techniques
BMI: body mass index
CVD: cardiovascular disease
I-Change model 2.0: Integrated Change Model 2.0
IM: intervention mapping
PRECEDE: Predisposing, Reinforcing, and Enabling Constructs in Educational/Environmental Diagnosis and Evaluation
RCT: randomized controlled trial

## References

[1] Puska P, Norrving B, Mendis S, Puska P, Norrving B, World Health Organization (2011). Global Atlas on Cardiovascular Disease Prevention and Control.

[2] de Silva D (2011). A review of the evidence considering whether it is worthwhile to support self-management. Evidence: Helping People Help Themselves.

[3] McGowan P (2005). Self-management: a background paper. http://bcsm.bluelemonmedia.com/uploads/Support%20for%20Health%20Professionals/Self-Management%20support%20a%20background%20paper%202005.pdf.

[4] Barlow J, Wright C, Sheasby J, Turner A, Hainsworth J (2002). Self-management approaches for people with chronic conditions: a review. Patient Educ Couns.

[5] Barnason S, Zimmerman L, Nieveen J, Schulz P, Young L (2012). Patient recovery and transitions after hospitalization for acute cardiac events: an integrative review. J Cardiovasc Nurs.

[6] Egberg L, Andreassen S, Mattiasson A (2012). Experiences of living with intermittent claudication. J Vasc Nurs.

[7] Satink T, Cup EH, Ilott I, Prins J, de Swart BJ, Nijhuis-van der Sanden MW (2013). Patients' views on the impact of stroke on their roles and self: a thematic synthesis of qualitative studies. Arch Phys Med Rehabil.

[8] Lorig KR, Ritter PL, Laurent DD, Plant K (2006). Internet-based chronic disease self-management: a randomized trial. Med Care.

[9] Sol BG, van der Bijl JJ, Banga J, Visseren FL (2005). Vascular risk management through nurse-led self-management programs. J Vasc Nurs.

[10] Sol Berna G BG, van der Graaf Y, van Petersen R, Visseren FL (2011). The effect of self-efficacy on cardiovascular lifestyle. Eur J Cardiovasc Nurs.

[11] Janssen V, De Gucht GV, Dusseldorp E, Maes S (2013). Lifestyle modification programmes for patients with coronary heart disease: a systematic review and meta-analysis of randomized controlled trials. Eur J Prev Cardiol.

[12] Ekeland AG, Bowes A, Flottorp S (2010). Effectiveness of telemedicine: a systematic review of reviews. Int J Med Inform.

[13] Clark RA, Inglis SC, McAlister FA, Cleland JG, Stewart S (2007). Telemonitoring or structured telephone support programmes for patients with chronic heart failure: systematic review and meta-analysis. BMJ.

[14] Neubeck L, Redfern J, Fernandez R, Briffa T, Bauman A, Freedman SB (2009). Telehealth interventions for the secondary prevention of coronary heart disease: a systematic review. Eur J Cardiovasc Prev Rehabil.

[15] Holland R, Battersby J, Harvey I, Lenaghan E, Smith J, Hay L (2005). Systematic review of multidisciplinary interventions in heart failure. Heart.

[16] Petty R, Cacioppo J (1986). Central and peripheral routes to attitude change. Communication and Persuasion.

[17] Bartholomew LK, Parcel GS, Kok G, Gottlieb NH, Fernández ME (2011). Planning Health Promotion Programs: An Intervention Mapping Approach Third Edition.

[18] Pocock SJ (1984). A practical approach. Clinical Trials.

[19] Green LW, Kreuter MW (2004). Health Program Planning: An Educational and Ecological Approach Fourth Edition.

[20] Brink E, Karlson BW, Hallberg LR (2006). Readjustment 5 months after a first-time myocardial infarction: reorienting the active self. J Adv Nurs.

[21] Brink E (2009). Adaptation positions and behavior among post--myocardial infarction patients. Clin Nurs Res.

[22] Wann-Hansson C, Hallberg IR, Klevsgård R, Andersson E (2005). Patients' experiences of living with peripheral arterial disease awaiting intervention: a qualitative study. Int J Nurs Stud.

[23] Kvigne K, Kirkevold M, Gjengedal E (2004). Fighting back--struggling to continue life and preserve the self following a stroke. Health Care Women Int.

[24] de Vries H, Kremers SP, Smeets T, Brug J, Eijmael K (2008). The effectiveness of tailored feedback and action plans in an intervention addressing multiple health behaviors. Am J Health Promot.

[25] de Bruin M, Kok G, Schaalma H, Hospers HJ (2007). Coding manual for behavioral change techniques.

[26] Craig P, Dieppe P, Macintyre S, Michie S, Nazareth I, Petticrew M, Medical Research Council Guidance (2008). Developing and evaluating complex interventions: the new Medical Research Council guidance. BMJ.

[27] Saunders RP, Evans MH, Joshi P (2005). Developing a process-evaluation plan for assessing health promotion program implementation: a how-to guide. Health Promot Pract.

[28] Health Council of the Netherlands (2006). Guidelines for a Healthy Diet.

[29] Arrindell WA, Sanavio E, Sica C (2002). Introducing a short form version of the Scale of Interpersonal Behaviour (s-SIB) for use in Italy. Psicoterapia Cognitiva e Comportamentale.

[30] Rollnick S, Miller WR, Butler C (2008). Motivational Interviewing in Health Care: Helping Patients Change Behavior.

[31] Brouwer W, Oenema A, Crutzen R, de Nooijer J, de Vries NK, Brug J (2008). An exploration of factors related to dissemination of and exposure to internet-delivered behavior change interventions aimed at adults: a Delphi study approach. J Med Internet Res.

[32] Brouwer W, Oenema A, Crutzen R, de Nooijer J, de Vries NK, Brug J (2009). What makes people decide to visit and use an internet‐delivered behavior‐change intervention?. Health Educ.

[33] Weinman J, Petrie KJ, Moss-morris R, Horne R (1996). The illness perception questionnaire: a new method for assessing the cognitive representation of illness. Psychol Health.

[34] VanderZee KI, Sanderman R, Heyink JW, de Haes H (1996). Psychometric qualities of the RAND 36-Item Health Survey 1.0: a multidimensional measure of general health status. Int J Behav Med.

[35] Hibbard JH, Mahoney ER, Stockard J, Tusler M (2005). Development and testing of a short form of the patient activation measure. Health Serv Res.

[36] ten Klooster PM, Oostveen JC, Zandbelt LC, Taal E, Drossaert CH, Harmsen EJ, van de Laar MA (2012). Further validation of the 5-item Perceived Efficacy in Patient-Physician Interactions (PEPPI-5) scale in patients with osteoarthritis. Patient Educ Couns.

[37] Horne R, Weinman J, Hankins M (1999). The beliefs about medicines questionnaire: the development and evaluation of a new method for assessing the cognitive representation of medication. Psychol Health.

[38] Morisky DE, Ang A, Krousel-Wood M, Ward HJ (2008). Predictive validity of a medication adherence measure in an outpatient setting. J Clin Hypertens (Greenwich).

[39] Pomerleau CS, Carton SM, Lutzke ML, Flessland KA, Pomerleau OF (1994). Reliability of the Fagerstrom Tolerance Questionnaire and the Fagerstrom Test for Nicotine Dependence. Addict Behav.

[40] Gual A, Segura L, Contel M, Heather N, Colom J (2002). Audit-3 and audit-4: effectiveness of two short forms of the alcohol use disorders identification test. Alcohol Alcohol.

[41] Craig CL, Marshall AL, Sjöström M, Bauman AE, Booth ML, Ainsworth BE, Pratt M, Ekelund U, Yngve A, Sallis JF, Oja P (2003). International physical activity questionnaire: 12-country reliability and validity. Med Sci Sports Exerc.

[42] van Lee L, Geelen A, van Huysduynen EJ, de Vries JH, van't Veer Veer P, Feskens EJ (2012). The Dutch Healthy Diet index (DHD-index): an instrument to measure adherence to the Dutch Guidelines for a Healthy Diet. Nutr J.

