# Supplementary material for: Self-Management Support Program for Patients With Cardiovascular Diseases: User-Centered Development of the Tailored, Web-Based Program Vascular View
Source: JMIR Res Protoc. 2017 Feb 8;6(2):e18. doi: 10.2196/resprot.6352 (PMC5322199; doi:10.2196/resprot.6352)
Supplement: Multimedia Appendix 1 [file resprot_v6i2e18_app1.pdf]

**Performance objective: patients …**	**Knowledge**	**Awareness**	**Risk Perception**
have insight into CVD and accompanying symptoms and consequences	have knowledge about CVD and accompanying symptoms and consequences	are aware of the importance of self managing CVD and accompanying symptoms and consequences	
cope with CVD and accompanying symptoms and consequences	have knowledge about CVD and accompanying symptoms and consequences	are aware of the importance of coping with CVD and accompanying symptoms and consequences	estimate the influence of coping with CVD and accompanying symptoms and consequences on quality of life
cope with (changed) sexuality and intimacy	have knowledge about coping with (changed) sexuality and intimacy	are aware of the possible influence of CVD on sexuality and intimacy	
cope with stress in daily life	have knowledge about coping with stress in daily life		
cope with fear and emotions related to CVD	have knowledge about possibly fear and emotions related to CVD	are aware of possibly fear and emotions related to CVD	
cope with pain related to CVD	have knowledge about possible pain related to CVD	are aware of possible pain related to CVD	estimate the importance of coping with pain related to CVD
adhere to medication instructions	have knowledge about medication	are aware of the importance of adherence to medication instructions	estimate the influence of adhering to medication instructions on CVD and accompanying symptoms and consequences
set boundaries	have knowledge of their own (changed) boundaries	are aware of the importance of setting boundaries	estimate the influence of setting boundaries on CVD and accompanying symptoms and consequences
adapt to changed circumstances	have knowledge of their own changed circumstances	are aware of the importance of adapting to changed circumstances	estimate the influence of adapting to changed circumstances on CVD and accompanying symptoms and consequences
ask for support from partner, relatives and social environment	have knowledge of the importance of asking for support from partner, relatives and social environment		
cope with changed roles in family, job	have knowledge to cope with changed	are aware of possibly changed roles in	

and/or society	roles in family, job and/or society	family, job and/or society	
are able to resume activities within their own possibilities	have knowledge of how to resume activities within their own possibilities		
eat healthy	have knowledge of healthy nutrition	are aware of the importance of eating healthy nutrition	estimate the influence of eating healthy in relation to CVD
are physically active	have knowledge of being physically active	are aware of the importance of being physically active	estimate the influence of being physically active in relation to CVD
refrain from tobacco use	have knowledge of the pros and cons of tobacco use	are aware of the importance of refraining from tobacco use	
refrain from (harmful) alcohol use	have knowledge of the pros and cons of alcohol use	are aware of the importance of refraining from alcohol use	
interact with health professionals	have knowledge of how to interact with health professionals	are aware of the importance and the possibilities of interacting with health professionals	
