# Supplementary material for: Self-Management Support Program for Patients With Cardiovascular Diseases: User-Centered Development of the Tailored, Web-Based Program Vascular View
Source: JMIR Res Protoc. 2017 Feb 8;6(2):e18. doi: 10.2196/resprot.6352 (PMC5322199; doi:10.2196/resprot.6352)
Supplement: Multimedia Appendix 2 [file resprot_v6i2e18_app2.pdf]

**Performance objective: patients …**	**Attitude**	**Self-efficacy**	**Subjective norm**	**Motivation (intention)**	**Habits**
have insight into CVD and accompanying symptoms and consequences					
cope with CVD and accompanying symptoms and consequences	have a positive attitude towards coping with CVD and accompanying symptoms and consequences	express confidence about coping with CVD and accompanying symptoms and consequences	cope with the reactions and expectations of important others when coping with CVD and accompanying symptoms and consequences	intend to cope with CVD and accompanying symptoms and consequences	routineously coping with CVD and accompanying symptoms and consequences
cope with (changed) sexuality and intimacy	have a positive attitude towards coping with (changed) sexuality and intimacy	express confidence about coping with (changed) sexuality and intimacy	cope with the reactions and expectations of important others when coping with (changed) sexuality and intimacy		
cope with stress in daily life	have a positive attitude towards coping with stress in daily life	express confidence about coping with stress in daily life	cope with the reactions and expectations of important others according to when coping with stress in daily life		
cope with fear and emotions related to CVD					
cope with pain related to CVD	have a positive attitude towards coping with pain related to CVD	express confidence about coping with pain related to CVD	cope with the reactions and expectations of important others when coping with pain related to CVD		
adhere to medication instructions	have a positive attitude towards adhering to	express confidence about adhering to medication	cope with the reactions and expectations of important		Make a habit of adhering to medication instructions

	medication instructions	instructions	others when adhering to medication instructions		
set boundaries	have a positive attitude towards setting boundaries	express confidence about setting boundaries	cope with the reactions and expectations of important others when setting boundaries		make a habit of setting boundaries
adapt to changed circumstances	have a positive attitude towards adapting to changed circumstances	express confidence about adapting to changed circumstances	cope with the reactions and expectations of important others when adapting to changed circumstances		make a habit of adapting changed circumstances
ask for support from partner, relatives and social environment	have a positive attitude towards asking support from partner, relatives and social environment	express confidence about asking support from partner, relatives and social environment	cope with reactions and expectations of important others when asking for support from partner, relatives and social environment		
cope with changed roles in family, job and/or society	have a positive attitude to cope with changed roles in family, job and/or society	express confidence about coping with changed roles in family, job and/or society	cope with reactions and expectations of important others according to possibly changed roles		
are able to resume activities within their own possibilities	have a positive attitude to resume activities within their own possibilities	express confidence about resuming activities within their own possibilities	cope with reactions and expectations of important others when resuming activities within their own possibilities		
eat healthy	have a positive attitude to eat healthy	express confidence about eating healthy	cope with reactions and expectations of important others when eating healthy	intend to eat healthily	make a habit of eating healthily
are physically	have a positive	express	cope with	intend to be	make a habit

active	attitude to be physically active	confidence about being physically active	reactions and expectations of important others when being physically active	physically active	of being physically active
refrain from tobacco use					
refrain from (harmful) alcohol use					
interact with health professionals	have a positive attitude to interacting with health professionals	express confidence in interacting with health professionals	cope with the reactions and expectations of important others according to interaction with health professionals		
			cope with the reactions and expectations of health professionals		
