# Supplementary figures and images for: Self-Management Support Program for Patients With Cardiovascular Diseases: User-Centered Development of the Tailored, Web-Based Program Vascular View
Source: JMIR Res Protoc. 2017 Feb 8;6(2):e18. doi: 10.2196/resprot.6352 (PMC5322199; doi:10.2196/resprot.6352)

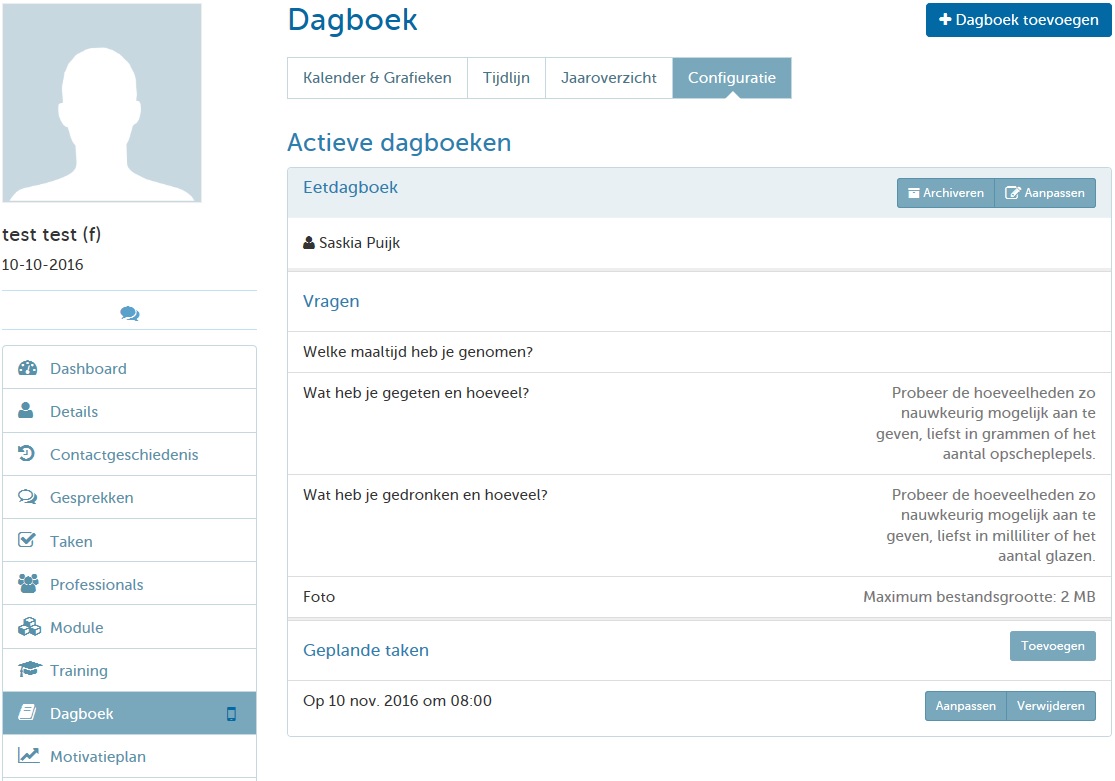

Supplement: Multimedia Appendix 3 [file resprot_v6i2e18_app3.jpg]

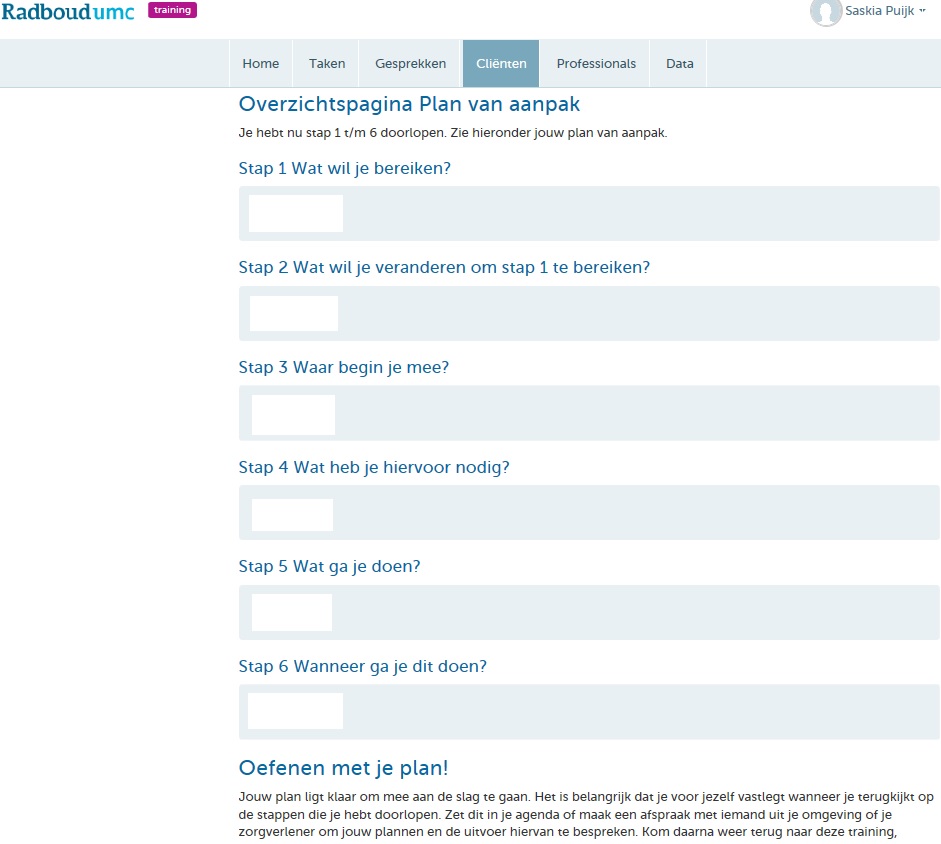

Supplement: Multimedia Appendix 4 [file resprot_v6i2e18_app4.jpg]

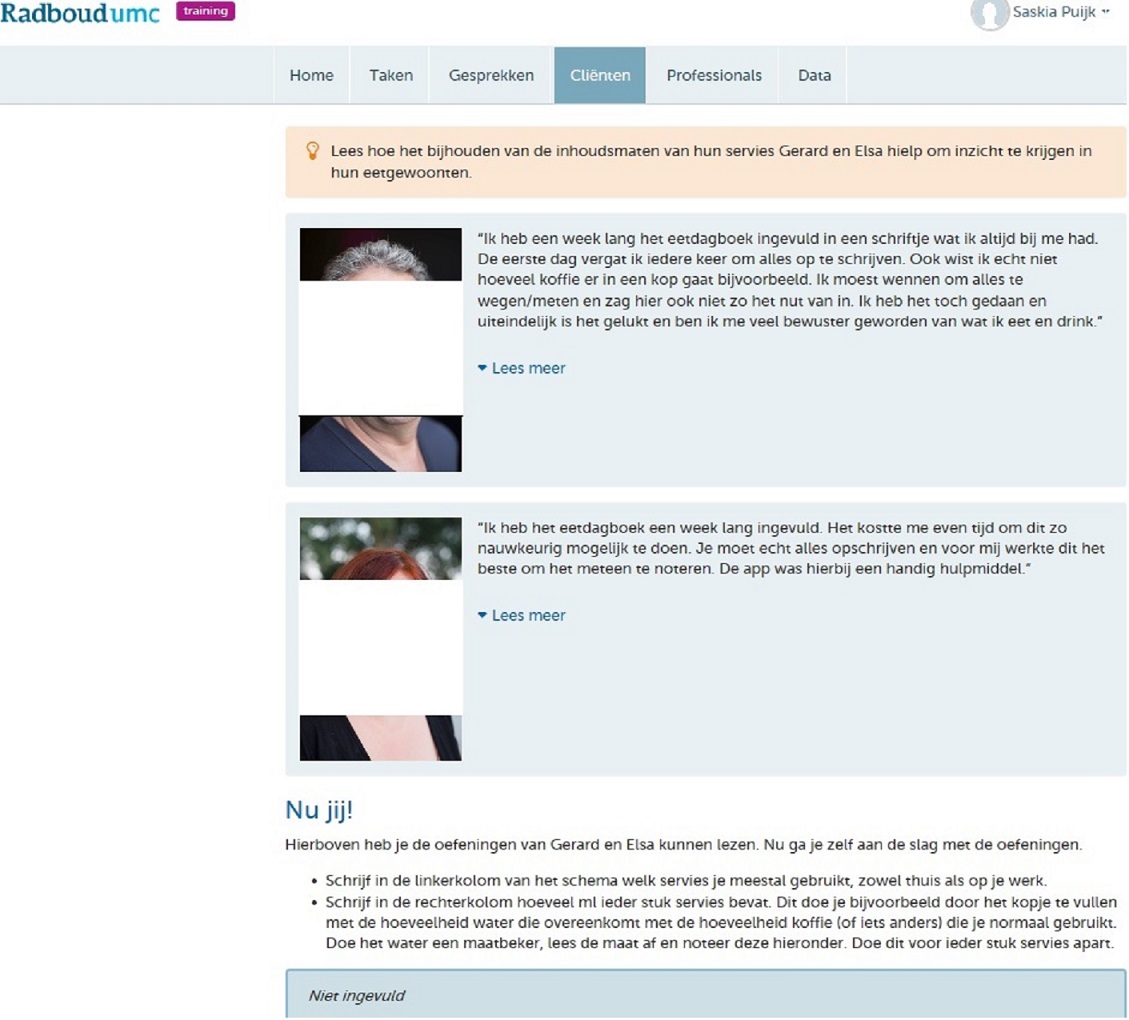

Supplement: Multimedia Appendix 5 [file resprot_v6i2e18_app5.jpg]
