# Supplementary material for: Self-Management Support Program for Patients With Cardiovascular Diseases: User-Centered Development of the Tailored, Web-Based Program Vascular View
Source: JMIR Res Protoc. 2017 Feb 8;6(2):e18. doi: 10.2196/resprot.6352 (PMC5322199; doi:10.2196/resprot.6352)
Supplement: Multimedia Appendix 6 [file resprot_v6i2e18_app6.pdf]

Determinants	Behavioral change techniques [25]	Parameters [17]	Examples of practical applications in 'Vascular View'
Awareness	Risk communication	None	Videos of role models (patients with CVD) presenting how they cope with risks, such as an unhealthy lifestyle.
	Self-monitoring of behavior	None	Digital self-report of eating and physical activity behavior by answering questions to get insight in the eating and physical activity diaries.
	Feedback: Delayed feedback of behavior	None	Completion of questionnaire about eating habits and receipt of tailored feedback, followed by the invitation to formulate goals in performing healthier behavior.
Attitude	Reevaluation of outcomes, self-evaluation	Stimulation of both cognitive and affective appraisal of self-image.	Digital self-report of eating and physical activity behavior by answering questions to get insight in the eating and physical activity diaries.
	Persuasive communication, belief selection	None	Receipt of tailored feedback about healthy food after completing a questionnaire about eating habits.<br><br>Videos of role models (patients with CVD) about the positive effects of changing behavior.
Intention	Specific goal setting	None	Accompanying goal setting by explaining how to set a goal (including role models), which is difficult, but available within the individual's skill level.
	Review of general and/or specific goals	None	Monitoring goals and the performed behavior based on a step-by-step plan, which will be filled in and evaluated by the patients themselves (8 steps about what to reach, how to change, where to start, what do you need, what are you going to do, when are you going to do this, evaluation and maintenance).
Self-efficacy	Modeling	Attention, remembrance, self-efficacy and skills,	Videos of role models (patients with CVD) presenting how they set, evaluate and maintain their goal-

		reinforcement of model, identification with model, coping model instead of mastery model.	behavior (e.g. how they became and stay more physically active).
	Set graded tasks, goal setting	The final behavior can be divided into easier but increasingly difficult sub-behaviors.	The ultimate goal of eating more healthy can be divided into smaller steps (e.g. this week I will eat two pieces of fruit every day). Patients are supported with examples and role models to reach their goals.
