# Supplementary material for: Self-Management Support Program for Patients With Cardiovascular Diseases: User-Centered Development of the Tailored, Web-Based Program Vascular View
Source: JMIR Res Protoc. 2017 Feb 8;6(2):e18. doi: 10.2196/resprot.6352 (PMC5322199; doi:10.2196/resprot.6352)
Supplement: Multimedia Appendix 7 [file resprot_v6i2e18_app7.pdf]

Course Coping with CVD	
Session	*Content*
1	General information about the content of the course.<br>Explanation of the benefits of following the course.
2	Information and exercises about CVD, its consequences on health and daily life, risks, and how to cope with CVD (resting, adaption of daily activities, own role within social environment, and using tools).
3	Information and exercises about how to cope with CVD (gaining insight into own behavior and reactions).<br>Coping with CVD in a good way and how to maintain this coping strategies in future.
Course Setting Boundaries in Daily Life	
Session	*Content*
1	General information about the content of the course.
2	Information and exercises about setting boundaries, asking for support/help and its benefits.<br>Information and exercises about positive and negative thoughts of setting boundaries and asking for support/help.
3	Information and exercises about setting boundaries by using verbal and nonverbal communication.<br>A test about how a patient defends oneself (Short form version of the Scale for Interpersonal Behavior (s-SIB)) [29].
4	Developing, practicing and evaluating an action plan to set boundaries in short- and long-term.
Course Lifestyle	
Session	*Content*
1	General information about healthy lifestyle (nutrition, physical activity, tobacco and alcohol use).<br>A short questionnaire about nutrition, weight, length, being physically active, tobacco and alcohol use, which aimed to advise a patient to perform other courses (Setting boundaries, Healthy nutrition and Being physically active in a healthy way).
2	Information and exercises to change lifestyle and to maintain the modifications.<br>Exercises for action planning to change lifestyle in short- and long-term.
3	General information about changing lifestyle into a healthier way, especially for tobacco use and harmful alcohol use.<br>Exercise for tobacco use, in which patients were asked to describe the pros and cons of risk behavior and to rate the personal importance of ceasing smoking and how confident they felt about success with cessation of the risk behavior [30].
4	General information about changing lifestyle to be more healthy, especially regarding alcohol use.<br>Exercise for alcohol use; same exercise as described for tobacco use in Session Three [30].
Course Healthy Nutrition	
Session	*Content*
1	General information about healthy nutrition, weight, body mass index (BMI) and waist circumference related to a healthy lifestyle.<br>Exercises in which patients assessed their own weight, height, BMI and waist circumference and received tailored feedback.
2	A questionnaire of 33 questions and the option to keep a diary about eating habits and food choices, including variation, regularity, snacks, fibers, vegetables, fruits, fat, salt, sweetening, caffeine, alcohol and losing weight. According the given answers, patients

	receive tailored feedback about their eating habits and the way they can change these habits if necessary.
3	An overview of the 33 answers in Session 2. The patient followed eight steps to make a personal plan to change, evaluate and maintain (including goal-setting) healthier eating behaviors.
Course Being Physically Active in a Healthy Way	
Session	Content
1	General information about being psychically active in a healthy way, related to a healthy lifestyle.<br>Information about possible perceived emotions about being physically active and how to deal with these emotions.
2	Patients gain insight into their physical activity habits by keeping a diary and answering additional questions, after which they received tailored feedback about their physical activity habits and the way they can change these habits if necessary.
3	An overview of the diary and answers given in Session 2. The patient followed eight steps to make a plan to change, evaluate and maintain healthier physical activity behaviors.
Course Interaction with Health Professionals	
Session	Content
1	General information and tips about communicating effectively with their health professional.
2	A checklist (including date, time, location, what to take and which questions to ask), which can be printed and taken to a consultation.
3	Information and exercises (practicing in real and evaluating) about how to be assertive during a consultation, how to communicate with and listen to your health professional.
4	Exercises for preparing, practicing and evaluating a consultation with a health professional.
